# miR-199a and miR-199b facilitate diffuse gastric cancer progression by targeting Frizzled-6

**DOI:** 10.1038/s41598-023-44716-0

**Published:** 2023-10-14

**Authors:** Soon Auck Hong, Sieun Lee, Jihye Park, Mineui Hong, Jung-Sook Yoon, Heejin Lee, Ji Hyun Lee, Seoree Kim, Hye Sung Won, Keunsoo Kang, Yoon Ho Ko, Young-Ho Ahn

**Affiliations:** 1 Department of Pathology, College of Medicine, Chung-Ang University, Seoul, 06974 Korea; 2 Department of Molecular Medicine and Inflammation-Cancer Microenvironment Research Center, College of Medicine, Ewha Womans University, 25 Magokdong-Ro 2-Gil, Gangseo-Gu, Seoul, 07804 Korea; 3 Uijeongbu St. Mary’s Hospital Clinical Research Laboratory, The Catholic University of Korea, Uijeongbu, 11765 Korea; 4 Department of Internal Medicine, Division of Oncology, College of Medicine, St. Mary’s Hospital, The Catholic University of Korea, 1021 Tongil-Ro, Eunpyeong-Gu, Seoul, 03312 Korea; 5 Cancer Research Institute, College of Medicine, The Catholic University of Korea, Seoul, 06591 Korea; 6 Department of Microbiology, College of Science and Technology, Dankook University, Cheonan, 31116 Korea

**Keywords:** Gastric cancer, Tumour biomarkers

## Abstract

Pathological markers that can monitor the progression of gastric cancer (GC) may facilitate the diagnosis and treatment of patients with diffuse GC (DGC). To identify microRNAs (miRNAs) that can differentiate between early and advanced DGC in the gastric mucosa, miRNA expression profiling was performed using the NanoString nCounter method in human DGC tumors. Ectopic expression of miR-199a and miR-199b (miR-199a/b) in SNU601 human GC cells accelerated the growth rate, viability, and motility of cancer cells and increased the tumor volume and weight in a mouse xenograft model. To study their clinicopathological roles in patients with GC, miR-199a/b levels were measured in human GC tumor samples using in situ hybridization. High miR-199a/b expression level was associated with enhanced lymphovascular invasion, advanced T stage, and lymph-node metastasis. Using the 3′-untranslated region (UTR) luciferase assay, Frizzled-6 (FZD6) was confirmed to be a direct target of miR-199a/b in GC cells. siRNA-mediated depletion of FZD6 enhanced the motility of SNU601 cells, and addback of FZD6 restored cancer cell motility stimulated by miR-199a/b. In conclusion, miR-199a/b promotes DGC progression by targeting FZD6, implying that miR-199a/b can be used as prognostic and diagnostic biomarkers for the disease.

## Introduction

Gastric cancer (GC) ranks as the fifth most prevalent cancer globally and stands as the fourth major contributor to cancer-related fatalities^1^. While surgery remains a common recommendation for localized GC, its effectiveness is confined primarily to patients diagnosed with early-stage disease.^2^*.* Recently, enhanced understanding of GC has led to new classifications: Epstein–Barr virus-positive, microsatellite unstable, chromosomally unstable, and genomically stable types^3^*.* Given the limited means for molecular subclassification, GC shows considerable clinicopathologic heterogeneity among tumors and an unpredictable clinical course in patients with similar clinical stages.

Despite their limited usefulness in guiding clinical treatment due to the molecular diversity of gastric cancer (GC), traditional morphology-based classification systems, such as Lauren’s classification, have continued to serve as important pathological tools^4^. In Lauren’s classification, GC can be categorized (according to the histological glandular formation of tumor cells) into diffuse-type GC (DGC), intestinal-type GC (IGC), and mixed-type GC^5^. Furthermore, DGC manifests distinctive morphological features of tumor cells with intracellular mucin and eccentric nuclei, known as signet ring cells.

DGC is not only characterized by morphological features of the tumor cells but also shows distinct clinicopathological characteristics, such as occurrence at a younger age and in the proximal stomach, and less association with chronic infection by *Helicobacter pylori*^5,6^. DGC represents approximately 30% of all GC cases and often exhibits more aggressive characteristics and poorer clinical outcomes than IGC^7^. In particular, advanced DGC (ADGC) is associated with a vicious prognosis, which could be caused by diffuse infiltration of the gastric wall and a strong potential for peritoneal seeding. However, early DGC (EDGC), in which the level of invasion is limited to the mucosa and/or submucosa, is associated with slow progression and better prognosis^8^. Although a discrepant prognosis between EDGC and ADGC has been noted, the morphology of the tumor cells is very similar^9^. Originally, the prediction of tumor nature is related to tumor cell morphology, which is usually evaluated in terms of grade and differentiation. Thus, new biomarkers to solve the contradiction between tumor cell morphology and tumor behavior in EDGC and ADGC are required for effectively treating patients with DGC.

MicroRNAs (miRNAs) are non-coding, single-stranded RNA molecules that are relatively short in length, typically measuring around 22–25 nucleotides, and they play a significant role in gene regulation. miRNAs function by binding to the 3´-untranslated regions (UTRs) of target mRNAs, which results in mRNA degradation or translational repression^10^. The dysregulation of miRNAs is commonly observed in various types of cancer, where numerous miRNAs exhibit altered expression levels in cancerous tissues compared to non-neoplastic tissues. These miRNAs regulate cancer-associated genes (either oncogenes or tumor-suppressor genes), which regulate cell proliferation, invasion, metastasis, differentiation, apoptosis, and drug resistance^11,12^.

In GC, miRNA signatures have been extensively studied and considered potential biomarkers in clinical practice. Plasma miR-421 has been suggested as a new biomarker for early GC^13^, and a serum miRNA signature including miR-1, miR-20, miR-27a, miR-34, and miR-423-5p has been proposed as a diagnostic marker for GC^14^. In addition, miRNA profiles can predict the prognosis and response to S-1 and doxorubicin in patients with GC^15–17^. Considering these studies, we hypothesize that certain miRNAs can be potential diagnostic and prognostic biomarkers capable of revealing the differences between ADGC and EDGC, hidden in morphological similarities. To test this hypothesis, we profiled miRNAs in DGC tumors using the NanoString nCounter method and found that miR-199a and miR-199b are strongly upregulated in ADGC. Our findings demonstrate that miR-199a and miR-199b promote the growth and migration of GC cells by targeting Frizzled-6 and thereby facilitating GC progression.

## Results

### miR-199a and miR-199b are upregulated in ADGC and promote the proliferation and migration of GC cells

**Figure 1:**
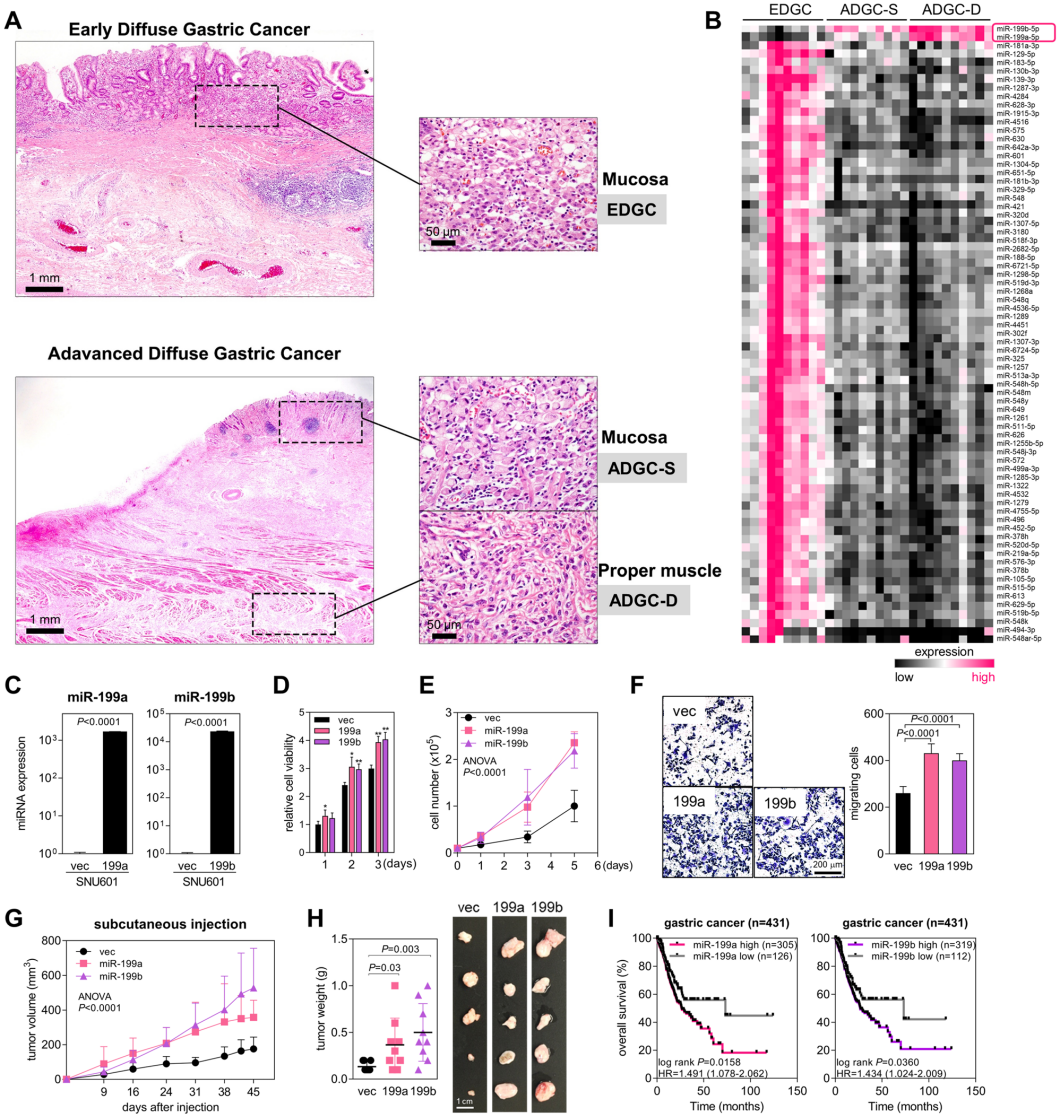
miR-199a and miR-199b are upregulated in ADGC and promote the growth and migration of GC cells. (**A**) Representative histologic images of diffuse gastric cancer (DGC). Paraffin sections of early DGC (EDGC) and advanced DGC (ADGC) were stained with hematoxylin and eosin (×12.5 magnification). Inserts (×400 magnification) are mucosa of EDGC, surface mucosa of advanced DGC (ADGC-S), and deeper mucosa (or proper muscle) of advanced DGC (ADGC-D). (**B**) Heatmap showing miRNA profiling with the NanoString nCounter method in EDGC, ADGC-S, and ADGC-D. Pink, increased expression level; black, decreased expression level (fold-change ≥ 2, *P* ≤ 0.01). (**C**) qRT-PCR of miR-199a or miR-199b in SNU601-vec, SNU601-miR199a, and SNU601-miR199b cells. Relative values to those of SNU601-vec are presented. Expression levels were normalized to *RNU6B* snoRNA levels. *P*, two-tailed Student’s *t*-test. (**D**, **E**) Cell growth analysis in SNU601-vec, SNU601-miR199a, and SNU601-miR199b cells. Cell viability (*D*) was measured with the WST-8 method (n = 4). The graph displays the relative cell viability compared to the control group (SNU601-vec), which was set to a value of 1.0. Cells (*E*) were also counted using an automated cell counter (n = 3). Data are mean + SD. *P < 0.05, **P < 0.01; two-tailed Student’s *t*-test. (**F**) Transwell migration assay with SNU601-vec, SNU601-miR199a, and SNU601-miR199b cells. Cells (1 × 10^5^/insert) were cultured in the upper wells for 24 h, and migrated cells were counted after staining with crystal violet. × 100 magnification. Data are mean + SD (n = 3). *P*, two-tailed Student’s *t*-test. (**G**) Tumor growth in BALB/c nude mice injected with SNU601-vec, SNU601-miR199a, and SNU601-miR199b cells. Tumor size was measured once a week for 6 weeks until mice were sacrificed, and tumor volume was calculated using the formula: volume = (width^2^ × length)/2. Data are mean + SD (n = 10). *P*, two-way ANOVA. (**H**) Tumors were removed from the mice and weighed at necropsy. Data are mean + SD (n = 10). *P*, two-tailed Student’s *t*-test. (**I**) Kaplan–Meier plots showing the overall survival rates of patients with GC in the TCGA database. Patients were divided into two groups (miR-199a/b_high and miR-199a/b_low) based on their miR-199a or miR-199b expression levels. *P*, log-rank test.

To identify miRNAs with expression patterns related to the progression of DGC, we performed NanoString nCounter assays on tumor tissues from the gastric mucosa of EDGC, surface mucosa of ADGC (ADGC-S), and deeper mucosa (muscularis propria) of ADGC (ADGC-D) (Fig. 1A and B). Numerous miRNAs showed differential expression patterns between EDGC and ADGC:74 miRNAs were downregulated, and miR-199a and miR-199b were significantly upregulated in ADGC (fold-change ≥ 2, *P* ≤ 0.05) compared with those in EDGC. miR-199a and miR-199b were previously reported to promote GC cell proliferation and migration through the regulation of mitogen-activated protein kinase kinase kinase 11 (MAP3K11)^18^ or hedgehog interacting protein (HHIP)^19^. Therefore, we selected miR-199a and miR-199b for further studies.

To investigate the roles of miR-199a and miR-199b in GC cells, miR-199a and miR-199b were overexpressed in SNU601 GC cells through lentiviral transduction (Fig. 1C). We then performed WST-8 assays using SNU601 cells transduced with miR-199a (SNU601-miR199a), miR-199b (SNU601-miR199b), or an empty vector (SNU601-vec) for three days (Fig. 1D). The results showed that the overexpression of miR-199a or miR-199b increased the viability of SNU601 GC cells. Cell counting assays for five days also confirmed that the growth of SNU601 cells increased when miR-199a or miR-199b was overexpressed (Fig. 1E). We also conducted EdU (5-ethynyl-2′-deoxyuridine) proliferation assays to examine the impact of miR-199a/b on the proliferation of SNU601 cells, which revealed that miR-199a/b significantly increased the proliferative activity of SNU601 cells (Supplementary Fig. 1A). To examine the role of miR-199a and miR-199b in cancer cell migration and invasion, we performed Transwell migration and invasion assays. SNU601-miR199a, SNU601-miR199b, or SNU601-vec cells were seeded in the upper wells, and cell culture media with 10% FBS was added to the bottom wells of Transwell plates. For the invasion assays, the upper wells were pre-coated with Matrigel. After 24 h of incubation, SNU601-miR199a and SNU601-miR199b migrated and invaded better than SNU601-vec did (Fig. 1F; Supplementary Fig. 1B). Next, to investigate the effect of miR-199a and miR-199b on tumor growth, SNU601-miR199a, SNU601-miR199b, or SNU601-vec was subcutaneously injected into BALB/c nude mice, and tumor growth was measured twice a week for 45 days. In both tumor volume and weight, tumors developed from SNU601-miR199a or SNU601-miR199b were bigger than those from SNU601-vec (Fig. 1G, H). These results suggested that miR-199a and miR-199b promote the growth, migration, invasion, and tumorigenesis of GC cells. In addition, high expression levels of miR-199a or miR-199b were significantly associated with a poor overall survival rate in patients with GC in The Cancer Genome Atlas (TCGA) data (Fig. 1I). Collectively, these results indicate that miR-199a and miR-199b promote GC progression.

### miR-199a and miR-199b inhibit GC cell adhesion

**Figure 2:**
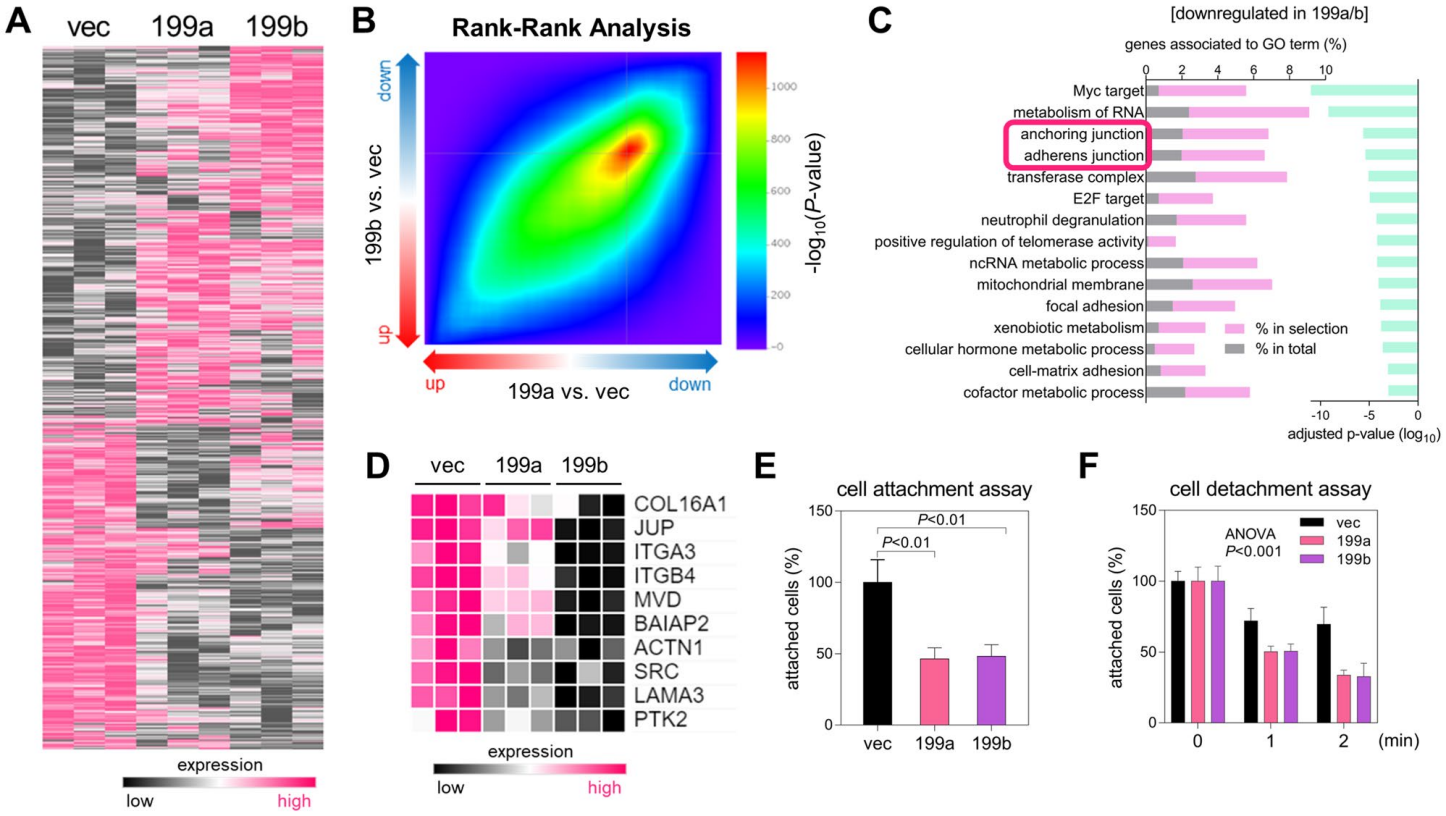
miR-199a and miR-199b suppress GC cell adhesion. (**A**) Heatmap showing mRNA sequencing results in SNU601-vec, SNU601-miR199a, and SNU601-miR199b cells. Pink, increased expression level; black, decreased expression level (fold-change ≥ 2, *P* ≤ 0.01). (**B**) Rank-rank hypergeometric overlap analysis of two profiling datasets: SNU601-vec versus SNU601-miR199a (x-axis; 199a vs. vec) and SNU601-vec versus SNU601-miR199b (y-axis; 199b vs. vec). Hypergeometric *P*-value is indicated in the color scale. (**C**) Gene ontology (GO) enrichment analysis of 484 genes downregulated in both SNU601-miR199a and SNU601-miR199b compared with SNU601-vec. Enriched GO terms were analyzed using Metascape (https://metascape.org). Genes associated with the GO terms are shown in the left bar graph for downregulated genes (% in selection, pink) and for the total set of 28,133 genes (% in total, grey). Adjusted *P*-values are represented in the right bar graph (green). (**D**) Heatmap showing the qRT-PCR results of cell adhesion-related genes in SNU601-vec, SNU601-miR199a, and SNU601-miR199b cells. Pink, increased expression level; black, decreased expression level. (**E**) Cell attachment assay with SNU601-vec, SNU601-miR199a, and SNU601-miR199b cells. Cells (3 × 10^5^/well) were seeded on collagen-coated 24-well plates, and non-adherent cells were removed by washing with PBS after 30 min. Attached cells were measured by crystal violet staining. Data are mean + SD (n = 3). *P*, two-tailed Student’s *t*-test. (**F**) Cell detachment assay with SNU601-vec, SNU601-miR199a, and SNU601-miR199b cells. Cells (1 × 10^5^/well) were cultured on 24-well plates for 24 h and then treated with trypsin/EDTA (0.025%/0.265 mM) for 1 or 2 min. Attached cells were measured by crystal violet staining. Data are mean + SD (n = 3). *P*, two-way ANOVA.

To identify the cellular signaling pathways regulated by miR-199a and miR-199b, we profiled global gene expression through RNA sequencing in SNU601-miR199a, SNU601-miR199b, and SNU601-vec cells (Fig. 2A). In the analysis of differentially expressed genes, SNU601-miR199a and SNU601-miR199b showed similar gene expression patterns which were quite different from that of SNU601-vec. In the rank-rank hypergeometric analysis, two gene expression profiles (199a vs. vec and 199b vs. vec) significantly overlapped among the downregulated genes (Fig. 2B). Gene ontology (GO) term analysis on the downregulated genes by both miR-199a and miR-199b revealed that these genes are associated to “anchoring junction”, “adherens junction”, and “focal adhesion” (Fig. 2C), and GO term analysis on the upregulated genes showed that they are associated with “eukaryotic translation elongation”, “positive regulation of DNA biosynthetic process”, and “negative regulation of apoptotic signaling pathway” (Supplementary Fig. 2A). Among the downregulated genes associated to “anchoring junction” and “adherens junction”, expression levels of the top 10 genes were measured by qRT-PCR in SNU601-miR199a and SNU601-miR199b (Fig. 2D; Supplementary Fig. 2B). As expected, most cell adhesion-related genes were downregulated in SNU601-miR199a and SNU601-miR199b cells compared to SNU601-vec, suggesting that miR-199a and miR-199b regulate cell–cell or cell–matrix adhesion. This result was supported by cell attachment and detachment assays: miR-199a and miR-199b decreased cell attachment to the collagen matrix (Fig. 2E) and facilitated detachment from the matrix in GC cells (Fig. 2F). These data suggest that miR-199a and miR-199b reduce the extent of cell–cell or cell–matrix adhesion, potentially promoting the dissociation and dissemination of GC cells from primary tumors.

### miR-199a and miR-199b promote GC cell growth and migration via targeting Frizzled-6 (FZD6)

**Figure 3:**
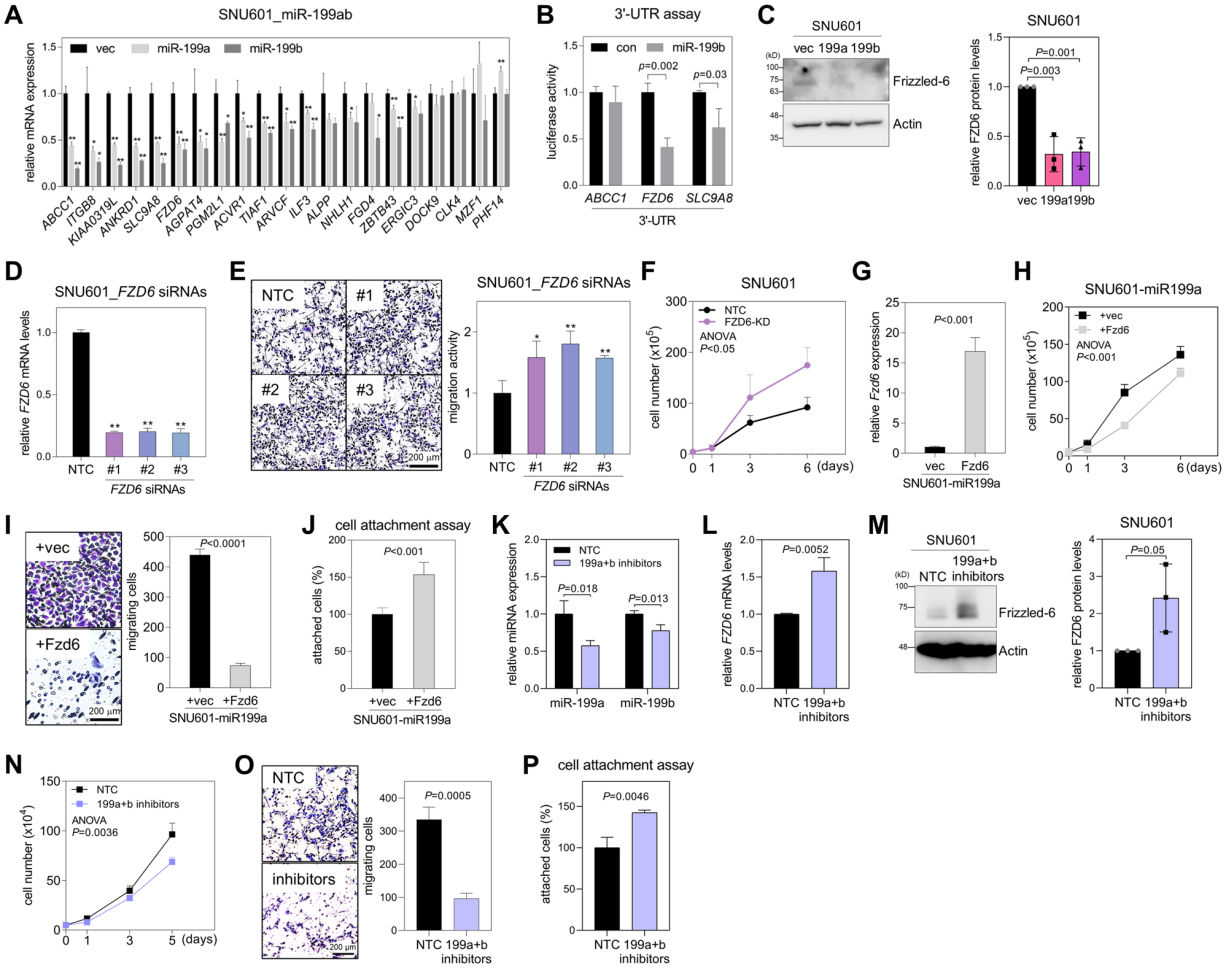
miR-199a and miR-199b promote GC cell growth and migration via targeting Frizzled-6. (**A**) qRT-PCR of candidate target genes of miR-199a/b in SNU601-vec, SNU601-miR199a, and SNU601-miR199b cells. Relative values to those of SNU601-vec are presented. Expression levels were normalized to *RPL32* mRNA levels. *P < 0.05, **P < 0.01; two-tailed Student’s *t*-test. (**B**) Luciferase reporter assay with 3′-UTRs of *ABCC1*, *FZD6*, and *SLC9A8*. 3′-UTRs containing putative miR-199a/b-binding sites (predicted using TargetScan) were cloned into a luciferase reporter vector (psiCHECK-2) and then co-transfected with the miR-199b mimic or control into 293T cells. Data are mean + SD (n = 3). *P*, two-tailed Student’s *t*-test. (**C**) Western blotting of Frizzled-6 in in SNU601-vec, SNU601-miR199a, and SNU601-miR199b cells. Actin was used as a loading control. Densitometric analysis of Western blot results (n = 3) is presented in the graph. (**D**) qRT-PCR of *FZD6* in SNU601 transfected with non-targeting control (NTC) or *FZD6* siRNAs (#1, #2, and #3). *P*, two-tailed Student’s *t*-test. (**E**) Transwell migration assay in SNU601 transfected with control (NTC) or *FZD6* siRNAs. Cells (1 × 10^5^/insert) were cultured in the upper wells for 24 h, and migrated cells were counted after staining with crystal violet. × 100 magnification. Data are mean + SD (n = 3). *P < 0.05, **P < 0.01; two-tailed Student’s *t*-test. (**F**) Cell growth analysis in SNU601 transfected with control (NTC) or *FZD6* siRNA #1 (FZD6-KD). Cells were counted using an automated cell counter. Data are mean + SD (n = 3). *P*, two-way ANOVA. (**G**) qRT-PCR of *FZD6* in SNU601-miR199a transfected with an empty vector (+ vec, pEGFP-N1) or murine Fzd6-expression vector (+ Fzd6). *P*, two-tailed Student’s *t*-test. (**H**) Cell growth analysis in SNU601-miR199a + vec and SNU601-miR199a + Fzd6. Cells were counted using an automated cell counter. Data are mean + SD (n = 3). *P*, two-way ANOVA. (**I**) Transwell migration assay in SNU601-miR199a + vec and SNU601-miR199a + Fzd6. Cells (1 × 10^5^/insert) were cultured in the upper wells for 24 h, and migrated cells were counted after staining with crystal violet. × 100 magnification. Data are mean + SD (n = 3). *P*, two-tailed Student’s *t*-test. (**J**) Cell attachment assay with SNU601-miR199a + vec and SNU601-miR199a + Fzd6. Cells (3 × 10^5^/well) were seeded on collagen-coated 24-well plates, and non-adherent cells were removed by washing with PBS after 30 min. Attached cells were measured by crystal violet staining. Data are mean + SD (n = 3). *P*, two-tailed Student’s *t*-test. (**K**, **L**) qRT-PCR of miR-199a, miR-199b (*K*), and *FZD6* mRNA (*L*) in SNU601 cells transfected with miR-199a and miR-199b inhibitors (199a + b inhibitors) or NTC. Data are mean + SD (n = 3). *P*, two-tailed Student’s *t*-test. (**M**) Western blotting of Frizzled-6 in in SNU601 cells transfected with miR-199a + b inhibitors or NTC. Actin was used as a loading control. Densitometric analysis of Western blot results (n = 3) is presented in the graph. (**N**) Cell growth analysis in SNU601 cells transfected with miR-199a + b inhibitors or NTC. Cells were counted using an automated cell counter. Data are mean + SD (n = 3). *P*, two-way ANOVA. (**O**) Transwell migration assay in SNU601 cells transfected with miR-199a + b inhibitors or NTC. × 100 magnification. Data are mean + SD (n = 3). *P*, two-tailed Student’s *t*-test. (**P**) Cell attachment assay with SNU601 cells transfected with miR-199a + b inhibitors or NTC. Data are mean + SD (n = 3). *P*, two-tailed Student’s *t*-test.

To elucidate the mechanism through which miR-199a and miR-199b promote GC cell growth and migration, we attempted to identify their target genes. Candidate target genes (n = 21) were selected using two approaches: (i) their mRNA expression levels were negatively correlated with those of miR-199a and miR-199b in TCGA-stomach adenocarcinoma data, and (ii) their mRNA levels were downregulated by miRNA-199a and miR-199b in the RNA sequencing data (Fig. 2A). The mRNA levels of these candidate target genes were measured by qRT-PCR, and most were downregulated in SNU601-miR199a and SNU601-miR199b compared with SNU601-vec (Fig. 3A). Among the downregulated genes, three genes (*ABCC1*, *FZD6*, and *SLC9A8*) were selected because they showed high target probability in the TargetScan database^20^. We performed luciferase reporter assays with the 3′-UTR of these three genes and confirmed that *FZD6* and *SLC9A8* were direct targets of miR-199a and miR-199b (Fig. 3B; Supplementary Fig. 3A). We selected *FZD6*, which encodes Frizzled-6, as a putative candidate because it is targeted by miR-199 in colorectal cancer^21^, skin keratinocytes^22^, and thymic epithelial cells^23^.

Overexpression of miR-199a and miR-199b led to a suppression of Frizzled-6 protein expression in SNU601 cells (Fig. 3C). As anticipated, the tumors derived from SNU601-miR199a and SNU601-miR199b cells exhibited elevated levels of miR-199a/b and reduced levels of *FZD6* mRNA (Supplementary Fig. 3B). To confirm whether miR-199a and miR-199b promote GC progression by targeting Frizzled-6, we transfected *FZD6*-specific siRNAs into SNU601 cells (SNU601-siFZD6) (Fig. 3D). *FZD6* knockdown increased migration (Fig. 3E) and growth of SNU601 cells (Fig. 3F), similar to the effects of miR-199 overexpression. *FZD6* knockdown had minimal to no effect on the expression levels of miR-199a and miR-199b (Supplementary Fig. 3C). Next, we restored Frizzled-6 to SNU601-miR199a or SNU601-miR199b cells (Fig. 3G; Supplementary Fig. 3D) and performed cell growth, Transwell migration, and cell attachment assays. Frizzled-6 addback attenuated cell growth and migration potentiated by miR-199a and miR-199b overexpression (Fig. 3H, I; Supplementary Fig. 3E and F) and restored cell attachment ability suppressed by miR-199a in SNU601 cells (Fig. 3J; Supplementary Fig. 3G). In contrast to *FZD6* knockdown, *SLC9A8* knockdown did not lead to any significant alterations in the activities of SNU601 cells (Supplementary Fig. 4). Next, we utilized inhibitors against miR-199a-5p and miR-199b-5p to silence miR-199a and miR-199b. The combined use of these inhibitors effectively reduced the cellular levels of both miR-199a and miR-199b (Fig. 3K), while concurrently increasing the expression of *FZD6* mRNA and Frizzled-6 protein (Fig. 3L, M). Furthermore, the inhibition of miR-199a and miR-199b resulted in the suppression of cell growth and migration (Fig. 3N, O) and the promotion of cell attachment in SNU601 cells (Fig. 3P). These results suggest that miR-199a and miR-199b promote cell proliferation and migration and inhibit cell attachment in GC cells by targeting Frizzled-6. Frizzled-6 has been identified as a negative regulator of the canonical Wnt/β-catenin signaling pathway^24^. Knockdown of *FZD6* resulted in an upregulation of WNT4, a promoter of stemness and tumorigenesis in gastric cancer cells^25^ (Supplementary Fig. 5A), and facilitated the translocation of β-catenin into the nucleus (Supplementary Fig. 5B). While further investigations are required to unveil the precise signaling mechanism, these findings strongly suggest that miR-199a/b-mediated suppression of Frizzled-6 can activate the Wnt/β-catenin signaling pathway, thereby contributing to the progression of gastric cancer.

### High expression levels of miR-199a or miR-199b correlate with a poor prognosis in patients with DGC

**Figure 4:**
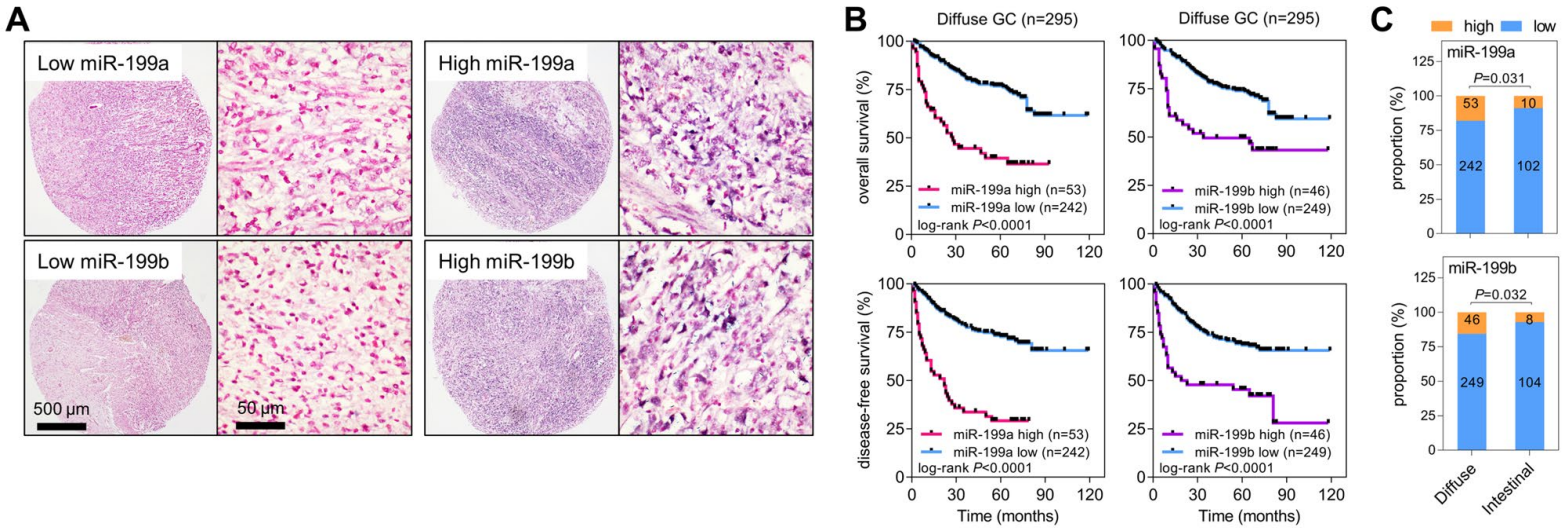
miR-199a and miR-199b are markers of poor prognosis in patients with DGC. (**A**) In situ hybridization of miR-199a or miR-199b in tissue microarrays of tumors from patients with DGC. Representative images of low/high miR-199a and miR-199b staining are presented. Original magnification × 40 (*left*) and × 400 (*right*). (**B**) Kaplan–Meier plots showing the overall survival (*top*) and disease-free survival rates (*bottom*) of patients with DGC. Patients were divided into two groups (miR-199a/b_high and miR-199a/b_low) based on their miR-199a (*left*) or miR-199b (*right*) expression levels. *P*, log-rank test. (**C**) In situ hybridization of miR-199a and miR-199b in tumor tissues from patients with DGC (n = 295) and intestinal GC (n = 112). Proportions of patients with high or low miR-199a/b expression levels are presented. *P*, Fisher’s exact test.

In the previous sections, miR-199a and miR-199b were shown to stimulate the proliferation and migration of GC cells. Therefore, we attempted to reveal the clinical relevance of these findings. Using probes specific to miR-199a or miR-199b, in situ hybridization was performed on tumor microarrays generated using tumor tissues from patients with DGC. The expression patterns of miR-199a and miR-199b were homogenous in tumor cells; thus, they were analyzed based on the intensity of expression (Fig. 4A). Patients were then divided into two groups (high and low) based on their miR-199a or miR-199b expression levels, and correlations between miR-199a/b levels and clinicopathological factors were evaluated (Tables 1 and 2). High expression levels of miR-199a or miR-199b were significantly associated with advanced GC and lymph-node metastasis. Furthermore, patients with high miR-199a or miR-199b expression levels showed worse survival rates than those with low miR-199a or miR-199b expression levels in both overall and disease-free survival analyses (Fig. 4B; Supplementary Table S2). To investigate the differential expression of miR-199a and miR-199b between the two histological types of GC, we measured miR-199a and miR-199b levels in 112 tumor tissues from patients with IGC (Tables 1 and 2). Although the number of IGC cases was small, we confirmed that there were no statistically significant differences in the proportions of early GC and advanced GC (*P* = 1) as well as the presence or absence of nodal metastasis (*P* = 0.983) between DGC and IGC groups (Supplementary Table S3). These factors, which could potentially affect the expression of miR-199a and miR-199b, were found to be comparable in both groups. The proportion of patients with high miR-199a and miR-199b expression levels was significantly higher in DGC than in IGC (Fig. 4C; Tables 1 and 2). In cases of IGC, we found no significant associations between the expressions of miR-199a and miR-199b and any clinicopathological parameters. These data suggest that miR-199a and miR-199b are markers for poor prognosis for patients with DGC.

**Table 1:** Correlations between miR-199a expression and clinicopathologic factors in DGC and IGC.

	miR-199a (DGC)		*P*-value	miR-199a (IGC)		*P*-value
	High (n = 53)	low (n = 242)		High (n = 10)	low (n = 102)	
Age			0.868			0.886
> 65	23 (43.4)	118 (48.8)		5 (50.0)	59(57.8)	
≤ 65	30 (56.6)	124 (51.2)		5 (50.0)	43(42.2)	
Sex			0.317			0.839
Male	35 (66.0)	179 (74.0)		6 (60.0)	70 (68.6)	
Female	18 (34.0)	63 (26.0)		4 (40.0)	32 (31.4)	
Location			0.919			0.925
Upper	9 (17.3)	45 (18.6)		1 (10.0)	9 (8.8)	
Mid	7 (13.5)	28 (11.6)		2 (20.0)	16 (15.7)	
Lower	36 (69.2)	169 (69.8)		7 (70.0)	77 (75.5)	
Lymphovascular invasion			0.183			0.156
Present	26 (50.9)	92 (38.0)		7 (70.0)	42 (41.2)	
Absent	27 (49.1)	150 (62.0)		3 (30.0)	60 (58.8)	
MMR			1			0.783
Deficiency	2 (3.8)	11 (4.5)		0 (0)	8 (7.8)	
Intact	51 (96.2)	231 (95.5)		10 (100)	94 (92.2)	
EBV			0.153			1
Positive	4 (7.5)	6 (2.5)		1 (10.0)	13 (12.7)	
Negative	49 (92.5)	236 (97.5)		9 (90.0)	89 (87.3)	
T stage			< 0.001*			0.416
1a and 1b (EGC)	2 (3.8)	124 (51.2)		6 (60.0)	42 (41.2)	
2, 3 and 4 (AGC)	51 (96.2)	118 (48.8)		4 (40.0)	60 (58.8)	
Lymph node metastasis			< 0.001*			0.791
Present	36 (67.9)	83 (34.3)		5 (50.0)	41 (40.2)	
Absent	17 (32.1)	159 (65.7)		5 (50.0)	61 (59.8)	

*DGC* diffuse-type gastric cancer, *IGC* intestinal-type gastric cancer, *EGC* early gastric cancer, *AGC* advanced gastric cancer.

**Table 2:** Correlations between miR-199b expression and clinicopathologic factors in DGC and IGC.

	miR-199b (DGC)		*P*-value	miR-199b (IGC)		*P*-value
	High (n = 46)	low (n = 249)		High (n = 8)	low (n = 104)	
Age			0.633			1
> 65	20 (43.5)	121 (48.6)		5 (62.5)	59 (56.7)	
≤ 65	26 (56.5)	128 (51.4)		3 (37.5)	45 (43.3)	
Sex			1			1
Male	33 (71.7)	181 (72.7)		5 (62.5)	71 (68.3)	
Female	13 (28.3)	68 (27.0)		3 (37.5)	33 (31.7)	
Location			0.956			0.254
Upper	8 (17.4)	46 (18.5)		2 (25.0)	8 (7.7)	
Mid	6 (13.0)	29 (11.7)		1 (12.5)	17 (16.3)	
Lower	32 (69.6)	173 (69.8)		5 (62.5)	79 (76.0)	
Lymphovascular invasion			0.095			0.460
Present	24 (52.2)	94 (37.8)		5 (62.5)	44 (42.3)	
Absent	22 (47.8)	155 (62.2)		3 (37.5)	60 (57.7)	
MMR			1			1
Deficiency	2 (4.3)	11 (4.4)		1 (12.5)	7 (6.7)	
Intact	44 (95.7)	238 (95.6)		7 (87.5)	97 (93.3)	
EBV			1			1
Positive	2 (6.4)	8 (3.2)		0 (0)	14 (13.5)	
Negative	44 (93.6)	241 (96.8)		8 (100)	90 (86.5)	
T stage			< 0.001*			1
1a and 1b (EGC)	8 (17.4)	118 (47.4)		3 (37.5)	45 (43.3)	
2, 3 and 4 (AGC)	38 (82.6)	131 (52.6)		5 (62.5)	59 (56.7)	
Lymph node metastasis			0.003*			0.873
Present	28 (60.9)	91 (36.5)		4 (50.0)	42 (40.4)	
Absent	18 (39.1)	158 (63.5)		4 (50.0)	62 (59.6)	

*DGC* diffuse-type gastric cancer, *IGC* intestinal-type gastric cancer, *EGC* early gastric cancer, *AGC* advanced gastric cancer.

## Discussion

DGC is characterized by neoplastic cells that are isolated or loosely arranged, without well-formed glands. Although the prognosis of ADGC is extremely poor compared with EDGC^26^, there is no significant difference between EDGC and ADGC, particularly in the gastric mucosa^9^. Because the prognosis of ADGC is extremely poor compared with that of EDGC, there is an urgent need to develop markers capable of distinguishing between EDGC and ADGC for the treatment of patients with DGC. Through miRNA profiling, we showed that miR-199a and miR-199b can be used as diagnostic markers for ADGC. Mechanistically, miR-199a and miR-199b promote GC cell growth and migration and suppress cell adhesion by targeting Frizzled-6, which results in GC progression.

Owing to their versatility and applicability, the clinical implications of miRNAs in GC development and progression have been extensively studied. In Epstein–Barr virus-associated GC, miR-200a and miR-200b are downregulated, which leads to the induction of ZEB1 and ZEB2, epithelial-mesenchymal transition-inducing transcription factors^27^. miR-101 is also downregulated in GC tissues and functions as a tumor suppressor^28^. In contrast, miR-181a is overexpressed in GC tissues compared to that in adjacent non-tumor tissues and is associated with tumor progression and distant metastasis. miR-181a exhibits oncogenic functions in GC cells by targeting Caprin-1, an RNA-binding protein^29^. miR-196a and miR-196b levels increased in GC and promoted GC cell migration and invasion by targeting radixin^30^. Similar to the results presented here, miR-199a and miR-199b have been previously reported to increase in GC tissues and promote GC cell proliferation, migration, and invasion through the regulation of mitogen-activated protein kinase kinase kinase 11 (MAP3K11) and hedgehog interacting protein (HHIP), respectively^18,19^. These miRNAs showed differential expression only in normal and GC tissues; however, we attempted to identify miRNAs that could more specifically differentiate between EDGC and ADGC. The expression levels of miR-199a and miR-199b were higher not only in GC tissues than in normal tissues^18,19^ but also in ADGC compared to EDGC.

miRNA profiling using the NanoString nCounter method revealed that more than 70 miRNAs were downregulated, and only two miRNAs, miR-199a and miR-199b, were upregulated in ADGC compared to EDGC. In particular, miR-199a and miR-199b were found to be much more highly expressed in the deeper layer than in the surface layer of gastric mucosa in GC tissues, suggesting that miR-199a and miR-199b are closely associated with the exacerbation of GC. Consistent with this, forced expression of miR-199a and miR-199b promoted GC cell growth and migration in vitro and enhanced tumorigenicity in vivo. Furthermore, miR-199a and miR-199b inhibited the cell–cell and cell–matrix adhesion of GC cells, which facilitated the dissociation and dissemination of GC cells.

Frizzled-6, a member of the Frizzled family, functions as a receptor for Wnt signaling proteins. Unlike other Frizzled proteins, Frizzled-6 lacks a PDZ-binding domain at its C-terminus and cannot activate canonical Wnt signaling. Instead, Frizzled-6 functions as a negative regulator of canonical Wnt/β-catenin signaling^24^ and transmits Wnt signals through non-canonical pathways, such as Wnt/planar cell polarity or Wnt/calcium signaling^31^. In GC, Frizzled-6 is targeted by miR-21, a well-known oncogenic miRNA^32^, and suppresses cancer cell proliferation and migration through opposing regulation of the canonical and non-canonical Wnt pathways^33^. Here, we also showed that Frizzled-6 is a direct target of miR-199a and miR-199b and inhibited GC cell growth and migration. miR-199a and miR-199b have been already described to target Frizzled-6 in colorectal cancer and thymic epithelial cells^21,23^. Subsequent studies on the effects of miR-199a and miR-199b on the non-canonical Wnt signaling pathway will help understand the pathogenesis of GC and develop potential therapeutics for patients with the disease.

Even among patients with histologically similar DGC, clinical outcomes are quite diverse. Patients with higher stromal gene expression have a poorer prognosis than those with lower stromal gene expression^34^. Recent proteomic analysis has further separated DGC into three subtypes: cell cycle, epithelial-mesenchymal transition, and immunological process enrichment subtypes, revealing the heterogeneity and diversity of DGC at the proteomic level^35^. Because cancer-associated miRNA biomarkers can be easily detected in tumor tissues, miRNAs have several potential advantages as biomarkers for predicting diverse clinical outcomes. Thus, miR-199a and miR-199b may be good candidates for prescreening DGC patients with poor prognoses.

In conclusion, miR-199a and miR-199b are selectively upregulated in ADGC and promote GC cell growth and migration by directly targeting Frizzled-6, which leads to GC progression. Based on the evidence presented here, miR-199a and miR-199b may be used as pathological biomarkers to discriminate between EDGC and ADGC. Moreover, miR-199a/b and Frizzled-6 are promising targets for developing therapies to prevent GC progression and metastasis.

## Methods

### Patients

The experimental protocols employed in this study received approval from the Institutional Review Boards (IRBs) of Soonchunhyang University Cheonan Hospital (approval number: 2019-01-006) and Chung-Ang University Hospital (approval number: 2015-010-421). All methods were conducted in compliance with the guidelines and regulations set forth by the respective IRBs. Tumor specimens from 20 patients with DGC (10 ADGC and 10 EDGC) who underwent radical gastrectomy at Soonchunhyang University Cheonan Hospital (Cheonan, Korea) were obtained for miRNA profiling. Informed consent was obtained from all participants. For miRNA in situ hybridization, surgically resected DGC (n = 295) and IGC (n = 112) tumors were retrieved from the Department of Pathology, Chung-Ang University Hospital (Seoul, Korea). Age, sex, survival time, and survival status were retrieved from the medical records. Pathological data regarding tumor location and size, lymphovascular and perineural invasion, primary tumor classification, and lymph-node metastasis were reviewed.

### Cell culture

SNU601 human GC cells were cultured in RPMI1640 (Welgene, Gyeongsan, Korea), and 293T cells were cultured in DMEM (Welgene) with 10% fetal bovine serum (FBS; HyClone, Logan, UT, USA) at 37 °C in the presence of 5% CO_2._ Cells were counted using an automated cell counter (Logos Biosystems, Anyang, Korea) after staining with trypan blue. For the cell viability assays, cells (1 × 10^4^/well) were cultured in 96-well plates, and cell viability was measured using a Quanti-Max WST-8 Cell Viability Assay Kit (BIOMAX, Seoul, Korea). For the cell proliferation assay, cells (4 × 10^5^/well) were cultured on coverslips in 6-well plates for 24 h, and cell proliferative activity was measured using Click-iT Plus EdU Imaging Kit (#C10639, Invitrogen; Waltham, MA, USA) according to the manufacturer’s protocol. For the Transwell migration assay, cells (1 × 10^5^/well) were cultured in the upper wells of hanging inserts (Corning, Corning, NY, USA) and allowed to migrate toward 10% FBS in the bottom wells. For the Transwell invasion assay, the upper wells were pre-coated with Matrigel (Corning). After 24 h of incubation, the migrated or invaded cells were fixed with 90% ethanol, stained with 0.1% crystal violet, photographed, and counted. For miR-199a and miR-199b overexpression, a genomic DNA region containing *MIR199A2* (chr1:172,144,314–172,144,763; 450 bp) or *MIR199B* (chr9:128,244,417–128,245,103; 687 bp) was isolated from 293T cell genomic DNA using PCR and ligated into the pLVX-Puro vector (Clontech, Mountain View, CA, USA). miR-199a_ or miR-199b_pLVX-Puro was introduced into SNU601 cells by lentiviral infection. Cells were infected with lentiviruses produced from 293T cells co-transfected with the lentiviral vectors, pMD2.G, and psPAX2 (gifts from Didier Trono; Addgene plasmids #12,259 and #12,260, respectively) for 2–3 days, and then selected with puromycin (2 μg/mL) for more than 2 weeks. *FZD6* siRNAs and miR-199a/miR-199b inhibitors were purchased from Bioneer (Daejeon, Korea) and transiently transfected into SNU601 cells using a TransIT-X2 Dynamic Delivery System (Mirus Bio, Madison, WI, USA). Murine *Fzd6* cDNA in the pEGFP-N1 vector (a gift from Bruce Beutler, Addgene plasmid #123,589)^36^ was stably transfected into SNU601-miR199a and SNU601-miR199b cells using Lipofector-EXT (AptaBio, Yongin, Korea).

### miRNA profiling in GC tumor samples

Twenty DGC samples (10 EDGC and 10 ADGC) were used for miRNA profiling. Unstained slides (×10) were cut from paraffin blocks in each case, and total miRNA was isolated from the paraffin sections using a miRNeasy FFPE Kit (Qiagen, Hilden, Germany). Following the evaluation of RNA quantity and quality, miRNA expression profiling was conducted by PhileKorea (Seoul, Korea) utilizing the nCounter Mouse v1.5 Assay kit from NanoString Technologies (Seattle, WA, USA). The miRNAs, which were tagged with oligonucleotides, were subjected to hybridization with miRNA code sets and processed in accordance with the manufacturer's instructions. The miRNA data were then collected and quantified using the nCounter Digital Analyzer, while the subsequent analysis was carried out using the nSolver software.

### Real-time quantitative reverse transcription PCR (qRT-PCR)

For the isolation of total RNA from SNU601 cells, the WelPrep Total RNA Isolation Reagent (Welgene) was utilized following the manufacturer's protocol. To analyze mRNA levels, qRT-PCR assays were performed using the BioFACT A-Star Real-time PCR Kit with SFCgreen I (BioFACT, Daejeon, Korea) after reverse transcription using the ELPIS RT Prime Kit (Elpis-Biotech, Daejeon, Korea). The mRNA levels were normalized to the *RPL32* mRNA. The qRT-PCR primer sequences employed in this study can be found in Supplementary Table S1. The quantification of miR-199a/b levels was carried out using the HB miR Multi Assay Kit (Heimbiotek, Seongnam, Korea) following the manufacturer's protocol, and the normalization was performed using the *RNU6B* snoRNA level. The quantification data were calculated using the ΔΔC_T_ method.

### Western blotting

Cell lysates were prepared using RIPA buffer (25 mM Tris–HCl pH 7.6, 150 mM NaCl, 1% NP40, 1% sodium deoxycholate, and 0.1% sodium dodecyl sulfate) containing protease inhibitors (Sigma-Aldrich; St. Louis, MO, USA). Protein samples were then separated by SDS-PAGE, transferred to PVDF membranes, and subsequently incubated with primary antibodies and HRP-conjugated secondary antibodies (Bio-Rad; Hercules, CA, USA). The protein bands were visualized using the Miracle-Star Western Blot Detection System from iNtRON Biotechnology (Seongnam, Korea). The following antibodies were used: Frizzled-6 (dilution 1:1000, #5158, Cell Signaling Technology; Danvers, MA, USA) and β-actin (dilution 1:5000, #BS6007M, Bioworld Technology; St. Louis Park, MN, USA).

### Mouse experiments

Prior to the commencement of the mouse studies, all planned experiments involving mice were subjected to submission and approval by the Institutional Animal Care and Use Committee (IACUC) of Ewha Womans University College of Medicine (Approval number: EUM20-036). The well-being and euthanasia of the mice adhered strictly to the guidelines established by the IACUC. Female BALB/c nude mice, 8 weeks old, were procured from Central Lab Animal (Seoul, Korea). SNU601 cells (2 × 10^6^ cells in 100 μL of serum-free RPMI1640) were subcutaneously injected into the flanks of the mice. Tumor growth was monitored twice a week, and after 6 weeks, the mice were humanely euthanized, and necropsies were performed to extract the primary tumors. The reporting in this manuscript follows the recommendations in the ARRIVE guidelines.

### RNA sequencing

Total RNA was extracted in triplicate from SNU601-vec, SNU601-miR199a, and SNU601-miR199b cells using the AccuPrep Universal RNA Extraction Kit (Bioneer). The RNA samples were then sent to Macrogen (Seoul, Korea) for RNA sequencing. Prior to library construction, the quantity and integrity of the total RNA were assessed. An RNA library was prepared using 1 µg of total RNA and the Illumina TruSeq Stranded mRNA Sample Prep Kit (Illumina, San Diego, CA, USA). Paired-end sequencing with read lengths of 2 × 100 bp was performed on an Illumina NovaSeq platform. The sequenced reads underwent quality-based trimming using Trim Galore (https://github.com/FelixKrueger/TrimGalore). Subsequently, the cleaned reads were aligned to the reference human genome (hg38) using STAR^37^. The abundance of known genes was estimated using StringTie^38^, employing the transcripts per kilobase million (TPM) method. Differential expression analysis was conducted using DESeq2^39^ with default parameters to identify differentially expressed genes (DEGs).

### Cell attachment and detachment assays

For cell attachment assays, cells (3 × 10^5^/well) were seeded on 24-well plates coated with 0.1 mg/mL of collagen (Corning) and allowed to attach for 30 min. For cell detachment assays, cells (1 × 10^5^/well) were cultured in 24-well plates for 24 h and detached using 0.025% trypsin/EDTA for 1 or 2 min. In both assays, the plates were washed with PBS to remove non-attached cells, and the attached cells were fixed with 90% ethanol and stained with crystal violet for 30 min. The dye was dissolved in 10% acetic acid, and the percentage of attachment was measured spectrophotometrically at 595 nm.

### 3′-UTR luciferase assay

The 3′-UTR regions of *ABCC1* (1734 bp), *FZD6* (1099 bp), and *SLC9A8* (1996 bp) that contain binding sites for miR-199a/b were amplified by PCR using genomic DNA from 293T cells. The amplified fragments were then cloned into the psiCHECK-2 vector (Promega, Madison, WI, USA). Prior to transfection, 293T cells were seeded at a density of 1 × 10^5^ cells per well in 24-well plates one day in advance. Transfection was carried out by introducing 500 ng of the 3′-UTR reporters using the TransIT-X2 reagent (Mirus Bio) in the presence or absence of miR-199a and miR-199b mimics (20 nM; Bioneer). Two days following transfection, luciferase activity was measured using the Luc-Pair Duo-Luciferase Assay Kit (GeneCopoeia, Rockville, MD, USA).

### miRNA in situ hybridization

Tissue microarrays containing 295 DGC and 112 IGC samples were used for in situ miRNA hybridization. In situ hybridization was performed using a miRCURY LNA miRNA ISH kit (Qiagen) with miR-199a-5p (5′-ACAGGTAGTCTGAACACT-3′) and miR-199b-5p (5′-AACAGATAGTCTAAACACT-3′) probes. The U6 and scramble miRNA probes provided in the kit were used as positive and negative controls, respectively. All probes were labeled with double digoxigenin (DIG) and denatured at 90 °C for 4 min before use. After deparaffinization and hydration, the slides were incubated with proteinase K (20 μg/mL) at 37 °C for 5 min (miR-199a-5p) or 15 min (miR-199b-5p). Slides were hybridized with probes against miR-199a-5p (50 nM, at 50 °C) and miR-199b-5p (125 nM at 40 °C) overnight in a hybridizer (ThermoBrite, Leica Biosystems, Richmond, IL). For probe detection, slides were incubated with sheep anti-DIG-alkaline phosphatase antibody (1:100; Roche, Mannheim, Germany) at room temperature for 1 h. The remaining steps were performed in accordance with the manufacturer’s instructions. The intensity of miR-199a and miR-199b expression was graded as follows: 0, no staining; 1, mild tumor cell staining; 2, moderate tumor cell staining; and 3, strong tumor cell staining. miR-199a and miR-199b expression levels were classified as low (0 and 1) and high (2 and 3), respectively.

### Statistical analysis

Data were analyzed by Student’s *t*-test, ANOVA, and log-rank test using GraphPad Prism (GraphPad Software; La Jolla, CA, USA) unless otherwise noted. Associations between miRNAs and clinicopathological parameters were evaluated using Fisher’s exact test or chi-square test, as needed. Overall survival (OS) was determined as the duration from the surgical intervention date to the date of death from any cause. Disease-free survival (DFS) was defined as the period from the surgical intervention date to either the date of initial recurrence or the last follow-up date. Survival analysis was performed using the Kaplan–Meier method, and the log-rank test was employed to identify statistically significant differences. Univariate and multivariate Cox proportional hazard analyses were conducted to identify independent prognostic factors for survival. Statistical significance was defined as a *P*-value of ≤ 0.05.

### Supplementary Information

Supplementary Information.

## Data Availability

The RNA sequencing data discussed in this publication have been deposited in NCBI's Gene Expression Omnibus (GEO) and are accessible through GEO Series accession number GSE227288 (https://www.ncbi.nlm.nih.gov/geo/query/acc.cgi?acc=GSE227288).
