# Supplementary material for: miR-199a and miR-199b facilitate diffuse gastric cancer progression by targeting Frizzled-6
Source: Sci Rep. 2023 Oct 14;13:17480. doi: 10.1038/s41598-023-44716-0 (PMC10576747; doi:10.1038/s41598-023-44716-0)

miR-199a and miR-199b facilitate diffuse gastric cancer progression by targeting Frizzled-6

Soon Auck Hong, Sieun Lee, Jihye Park, Mineui Hong, Jung-Sook Yoon, Heejin Lee, Ji Hyun Lee, Seoree Kim, Hye Sung Won, Keunsoo Kang, Yoon Ho Ko, Young-Ho Ahn

Supplementary Table S1. qRT-PCR primer sequences used in this study.

**genes**	**forward (5´→3´)**	**reverse (5´→3´)**
RPL32	ATCAGGCACCAGTCAGACCGAT	GTTGCTCCCATAACCGATGTTGG
BAIAP2	AGAGCAAGCTCAGCGACTCCTA	AGCGAGGCAGAGTCTTGTTCTC
ACTN1	CAGGACCGTGTGGAGCAGATTG	CAGATTGTCCCACTGGTCACAG
JUP	ACCAGCATCCTGCACAACCTCT	GGTGATGGCATAGAACAGGACC
SRC	GGACTAAGCAACAGGTGTTTCAAG	ACTGGAGGACTTGAGCCAACAG
COL16A1	AACAGTGAGGGAGATCCTGGCT	CAACAGCACCAGGAAAACCTGG
PTK2B	GCCTTATGACGAAATGCTGGGC	CCTGTCTTCTGGACTCCATCCT
ITGB4	AGGATGACGACGAGAAGCAGCT	ACCGAGAACTCAGGCTGCTCAA
ITGA3	GCCTGACAACAAGTGTGAGAGC	GGTGTTCGTCACGTTGATGCTC
MVD	AAGCGCGATGAAGAGCTGGTTC	TCCTCGGTGAAGTCCTTGCTGA
LAMA3	TAGAGGAAGCCTCTGACACAGG	CCGATAGTATCCAGGGCTACAAC
ABCC1	CCGTGTACTCCAACGCTGACAT	ATGCTGTGCGTGACCAAGATCC
ITGB8	CTGTTTGCAGTGGTCGAGGAGT	TGCCTGCTTCACACTCTCCATG
KIAA0319L	CTTTGGGAACCAGAGCACTGATG	GTGTAGTCTCCTTCTTGCATCGC
ANKRD1	CGACTCCTGATTATGTATGGCGC	GCTTTGGTTCCATTCTGCCAGTG
SLC9A8	AAGGCACACCGCAGGAACAAGA	TAAGGTCCTGCCGCCTGATGTA
FZD6	GGCAGTGTATCTGAAAGTGCGC	GATGTGGAACCTTTGAGGCTGC
AGPAT4	TCCGAACGCTTTGGGCTGTTAG	GCGAACAGAAGACCATCTCGGT
PGM2L1	CTAGCCACAGATCCTGATGCAG	CCACCATCCAAACAAAGCTGCC
ACVR1	GACGTGGAGTATGGCACTATCG	CACTCCAACAGTGTAATCTGGCG
TIAF1	GTTGAGCAAGCCTACGCAGACA	CAGTGCTTGGATCAGCCCTGAA
ARVCF	GCAGAAAGATGGTTGGACCAAGG	CAAGGCTCTTGTCCACCAGTGG
ILF3	CCTGACAAAGCACGGCAAGAAC	CCAGCACCTTGGAACTTCTGTC
ALPP	CAACGAGGTCATCTCCGTGATG	TACCAGTTGCGGTTCACCGTGT
NHLH1	CCCGACAAGAAGCTCTCCAAGA	CTAAGCAGTGAGCGGTGGAAAG
FGD4	GAGGCTCGTTTCCAGCAGAGAT	GGTGCCAATTTCTGAAGGATGTC
ZBTB43	TGAGCACGGAAATGGCAAGCCA	GATCCAGCGTTTGTGAGCCATG
ERGIC3	GCTATGGTGCTGAGGCAGAAGA	CTCCTGCATCTTCTGGCTGAAG
DOCK9	TCCAGAGGCATCTAAGAACGCG	GGTCTCCAGGAGCAAAACAGCT
CLK4	GCTATCGTGGAAGTCACAAGCG	CTCGCTCATTCAAGGACCTTGC
MZF1	GCAGGTGAAAGAGGAGTCAGAG	GTCCAGCACGGTCCCACATCT
PHF14	GATAGGTTAGACAGAAAGTGGAAG	CTTTGGCACGATACTGCTGAAGC

Supplementary Table S2. Univariate and multivariate analyses of overall and disease-free survival in 169 patients with ADGC

**Overall survival**	Univariate (*P*-value)	Multivariate (*P*-value)	HR	95% CI
**high miR-199a**	0.003	<0.001*	2.172	1.387-3.400
Sex (female *vs* male)	0.466			
Age (>65 *vs* ≤65)	0.046	0.075	1.469	0.963-2.240
T stage (4 *vs* 2, 3)	<0.001*	<0.001*	3.781	2.449-5.837
Lymph node metastasis	0.008	0.007	1.873	1.182-2.967
Lymphovascular invasion	0.009	0.550	1.195	0.666-2.142
EBV (positive *vs* low)	0.098			
MMR status (loss *vs* intact)	0.070			

**Disease-free survival**	Univariate (*P*-value)	Multivariate (*P*-value)	HR	95% CI
**high miR-199a**	0.001	<0.001*	2.338	1.538-3.556
Sex (female *vs* male)	0.388			
Age (>65 *vs* ≤65)	0.011	0.010*	1.684	1.132-2.505
T stage (4 *vs* 2, 3)	<0.001	<0.001*	2.318	1.529-3.514
Lymph node metastasis	0.002	0.001*	2.012	1.317-3.073
Lymphovascular invasion	0.010	0.138	1.161	0.682-1.976
EBV (positive *vs* low)	0.254			
MMR status (loss *vs* intact)	0.066			

**Overall survival**	Univariate (*P*-value)	Multivariate (*P*-value)	HR	95% CI
**high miR-199b**	0.008	0.008*	1.898	1.757-3.062
Sex (female *vs* male)	0.466			
Age (>65 *vs* ≤65)	0.046	0.053	1.515	0.994-2.309
T stage (4 *vs* 2, 3)	<0.001*	<0.001*	3.750	2.431-5.783
Lymph node metastasis	0.009	0.049*	1.5775	1.001-2.486
Lymphovascular invasion	0.008	0.484	1.238	0.682-2.249
EBV (positive *vs* low)	0.098			
MMR status (loss *vs* intact)	0.070			

**Disease-free survival**	Univariate (*P*-value)	Multivariate (*P*-value)	HR	95% CI
**high miR-199b**	0.008	0.004*	1.927	1.229-3.021
Sex (female *vs* male)	0.388			
Age (>65 *vs* ≤65)	0.011	0.010*	1.676	1.129-2.488
T stage (4 *vs* 2, 3)	<0.001	<0.001*	2.361	1.560-3.573
Lymph node metastasis	0.002	0.011*	1.719	1.120-2.616
Lymphovascular invasion	0.010	0.536	1.187	0.959-2.594
EBV (positive *vs* low)	0.254			
MMR status (loss *vs* intact)	0.099			

Supplementary Table S3. Comparison between DGC and IGC patient groups. DGC, diffuse-type gastric cancer; IGC, intestinal-type gastric cancer; EGC, early gastric cancer; AGC, advanced gastric cancer.

	AGC	EGC	*P*-value	lymph node metastasis		*P*-value
				present	absent	
DGC	169 (42.7)	126 (57.3)	1	119 (40.3)	176 (59.7)	0.983
IGC	64 (42.9)	48 (57.1)		46 (41.1)	66 (58.9)	

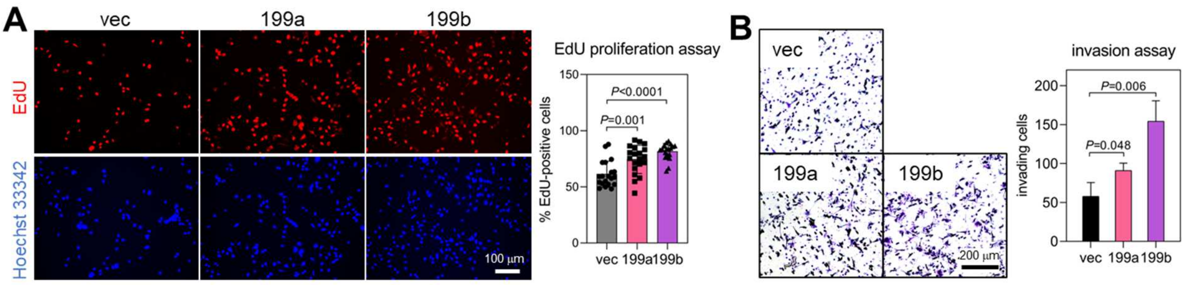

Supplementary Figure 1. miR-199a and miR-199b promote the proliferation and invasion of GC cells.

A. Cell proliferation analysis in SNU601-vec, SNU601-miR199a, and SNU601-miR199b cells. Cell proliferative activity was measured with the Click-iT Plus EdU Imaging Kit. Cells were incubated with EdU (5-ethynyl-2'-deoxyuridine) for 3 h, and EdU-incorporated DNA was labeled with Alexa Fluor 594 (red) through the click reaction. Total cells were stained with Hoechst 33342 (blue), a nuclear staining dye. The graph displays the percentage of EdU-positive cells among total cells (Hoechst 33342-positive cells). Data are mean + SD (n = 20). *P*, two-tailed Student's t-test.

B. Transwell invasion assay with SNU601-vec, SNU601-miR199a, and SNU601-miR199b cells. Cells (1×10^5/insert) were cultured in the upper wells which were pre-coated with Matrigel. After 24 h, invaded cells were counted after staining with crystal violet. ×100 magnification. Data are mean + SD (n = 3). *P*, two-tailed Student's t-test.

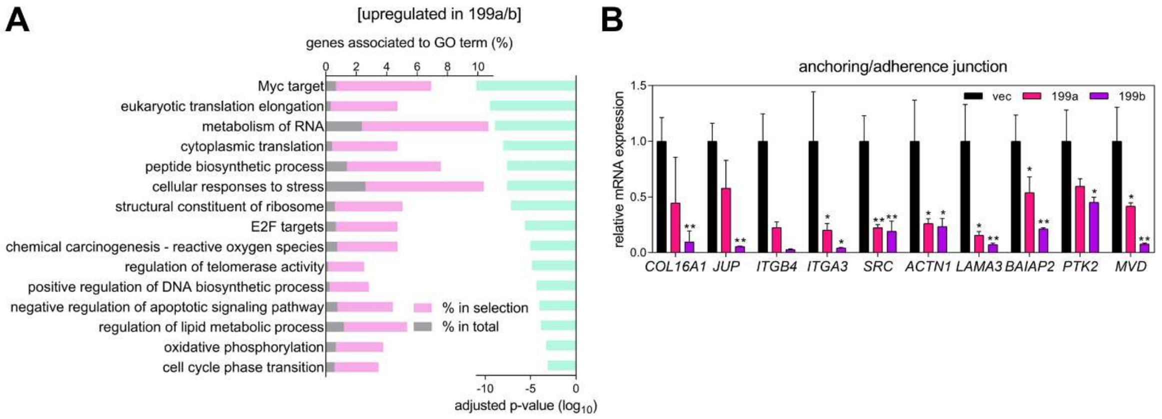

Supplementary Figure 2. miR-199a and miR-199b regulate global gene expression related to cell growth, proliferation, and adhesion.

A. Gene ontology (GO) enrichment analysis of 3318 genes upregulated in both SNU601-miR199a and SNU601-miR199b compared with SNU601-vec. Enriched GO terms were analyzed using Metascape (https://metascape.org). Genes associated with the GO terms are shown in the left bar graph for upregulated genes (% in selection, pink) and for the total set of 30,244 genes (% in total, grey). Adjusted *P*-values are represented in the right bar graph (green).
B. qRT-PCR of cell adhesion-related genes in SNU601-vec, SNU601-miR199a, and SNU601-miR199b cells. Relative values to those of SNU601-vec are presented. Expression levels were normalized to *RPL32* mRNA levels. $^*P < 0.05$, $^{**}P < 0.01$; two-tailed Student’s *t*-test.

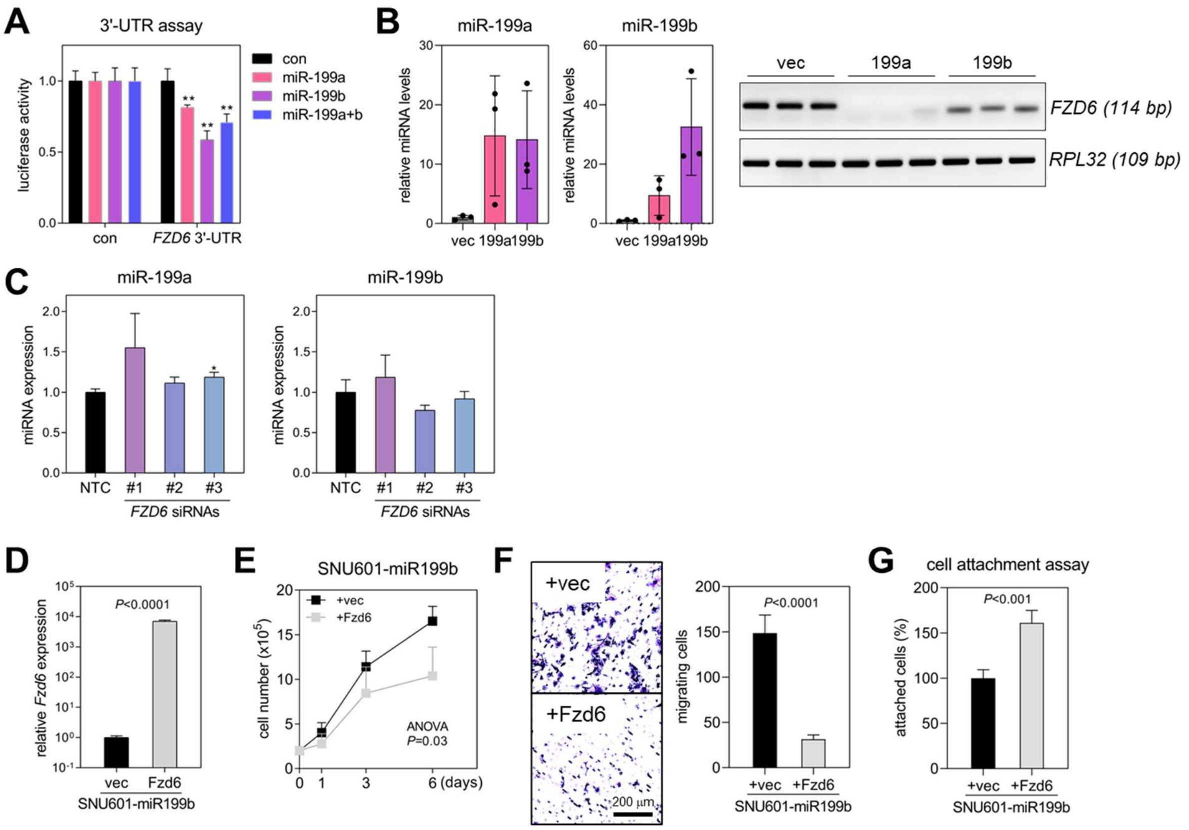

Supplementary Figure 3. miR-199a and miR-199b promote GC cell growth and migration via targeting Frizzled-6.

A. Luciferase reporter assay with *FZD6* 3′-UTR. Luciferase reporter vectors (psiCHECK-2) were co-transfected with the miR-199a/miR-199b mimics or control into 293T cells. Data are mean + SD (n = 3). *P*, two-tailed Student's *t*-test.
B. qRT-PCR of miR-199a and miR-199b (*left graphs*) and RT-PCR of *FZD6* mRNA (*right*) in tumors formed in nude mice after injection of SNU601-vec, SNU601-miR199a, and SNU601-miR199b cells. *RPL32* was used as control.
C. qRT-PCR of miR-199a and miR-199b in SNU601 transfected with non-targeting control (NTC) or FZD6 siRNAs (#1, #2, and #3). Data are mean + SD (n = 3). *P*, two-tailed Student's t-test.
D. qRT-PCR of *FZD6* in SNU601-miR199b transfected with an empty vector (+vec, pEGFP-N1) or murine Fzd6-expression vector (+Fzd6). Data are mean + SD (n = 3). *P*, two-tailed Student's t-test.
E. Cell growth analysis in SNU601-miR199b+vec and SNU601-miR199b+Fzd6. Cells were counted using an automated cell counter. Data are mean + SD (n = 4). *P*, two-way ANOVA.
F. Transwell migration assay in SNU601-miR199b+vec and SNU601-miR199b+Fzd6. Cells (1×10^5/insert) were cultured in the upper wells for 24 h, and migrated cells were counted after staining with crystal violet. ×100 magnification. Data are mean + SD (n = 3). *P*, two-tailed Student's *t*-test.
G. Cell attachment assay with SNU601-miR199b+vec and SNU601-miR199b+Fzd6. Cells ($3\text{x}10^5$/well) were seeded on collagen-coated 24-well plates, and non-adherent cells were removed by washing with PBS after 30 min. Attached cells were measured by crystal violet staining. Data are mean + SD (n = 3). *P*, two-tailed Student's *t*-test.

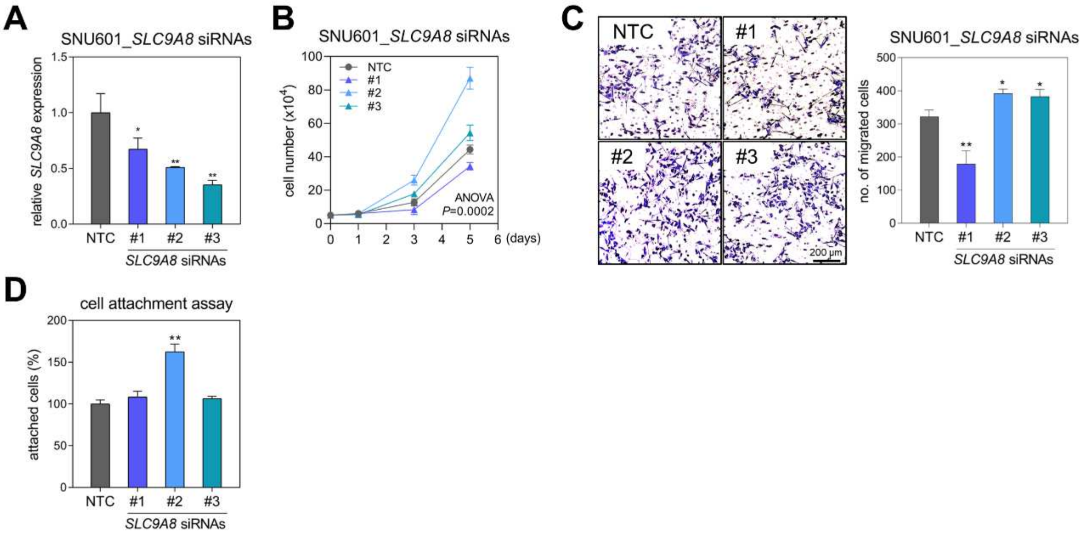

Supplementary Figure 4. *SLC9A8* knockdown causes minimal changes in the activities of SNU601 cells.

A. qRT-PCR of *SLC9A8* in SNU601 transfected with non-targeting control (NTC) or *SLC9A8* siRNAs (#1, #2, and #3). *P*, two-tailed Student's *t*-test.
B. Cell growth analysis in SNU601 transfected with control (NTC) or *SLC9A8* siRNAs (#1, #2, and #3). Cells were counted using an automated cell counter. Data are mean + SD (n = 3). *P*, two-way ANOVA.
C. Transwell migration assay in SNU601 transfected with control (NTC) or *SLC9A8* siRNAs (#1, #2, and #3). Cells (1×10^5/insert) were cultured in the upper wells for 24 h, and migrated cells were counted after staining with crystal violet. ×100 magnification. Data are mean + SD (n = 3). *$P < 0.05$, **$P < 0.01$; two-tailed Student's *t*-test.
D. Cell attachment assay in SNU601 transfected with control (NTC) or *SLC9A8* siRNAs (#1, #2, and #3). Cells ($3x10^5$/well) were seeded on collagen-coated 24-well plates, and non-adherent cells were removed by washing with PBS after 30 min. Attached cells were measured by crystal violet staining. Data are mean + SD (n = 3). **$P < 0.01$; two-tailed Student's *t*-test.

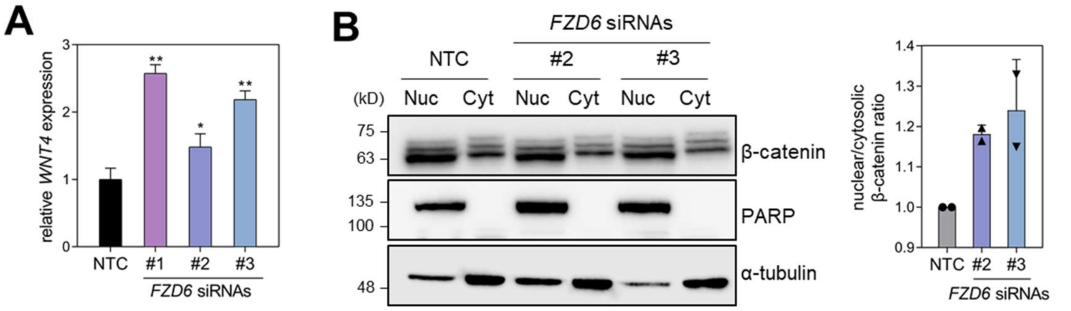

Supplementary Figure 5. *FZD6* knockdown facilitates the activation of Wnt/β-catenin signaling.

A. qRT-PCR of *WNT4* in SNU601 transfected with non-targeting control (NTC) or *FZD6* siRNAs (#1, #2, and #3). *P*, two-tailed Student's *t*-test.
B. Western blot of β-catenin in nuclear and cytosolic fractions from SNU601 transfected with non-targeting control (NTC) or *FZD6* siRNAs (#2 and #3). PARP was used as a nuclear marker, and α-tubulin was used as a cytosolic marker. The graph shows the ratios of nuclear to cytosolic β-catenin levels.

Original blots - Figure 3C

The blots were cut prior to hybridization with antibodies.

Digital Western blot images were obtained using the ChemiDoc system.

Figure 3C

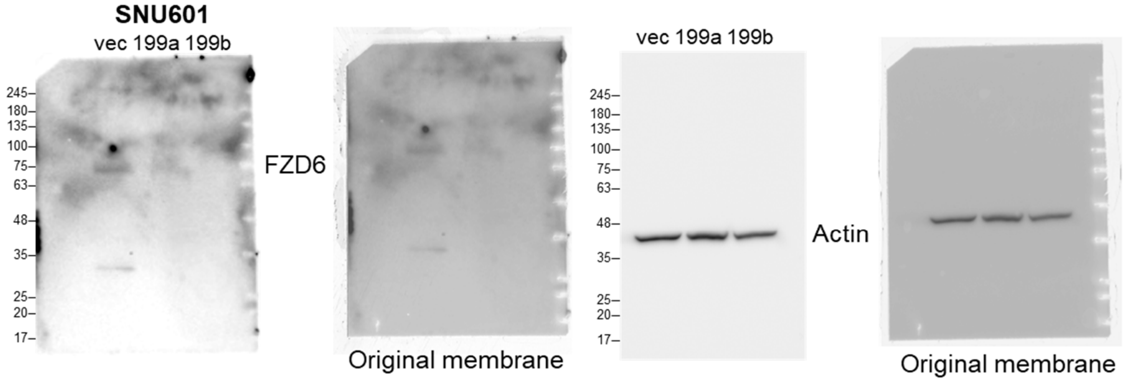

Figure 3C

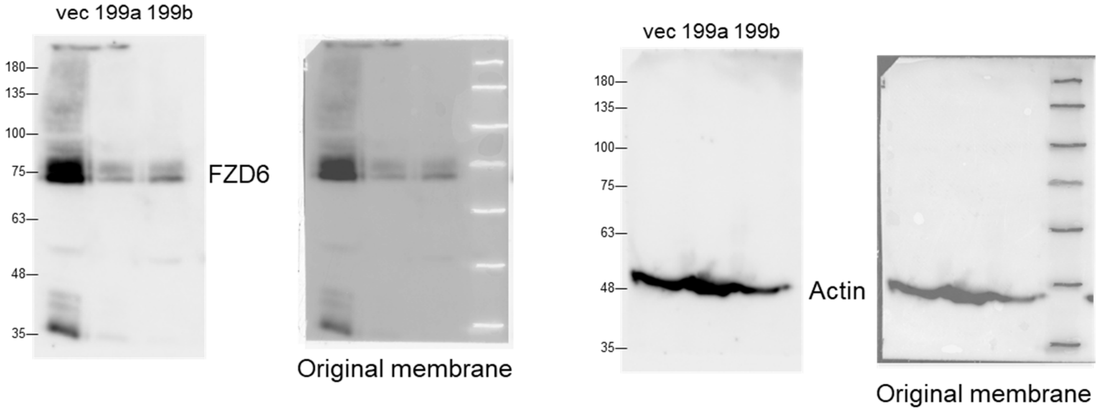

Figure 3C

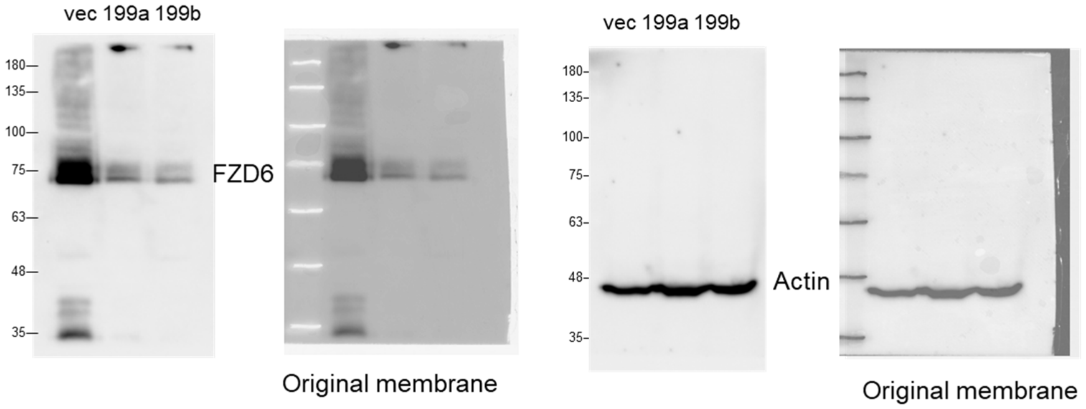

Original blots - Figure 3M

The blots were cut prior to hybridization with antibodies.

Digital Western blot images were obtained using the ChemiDoc system.

Figure 3M

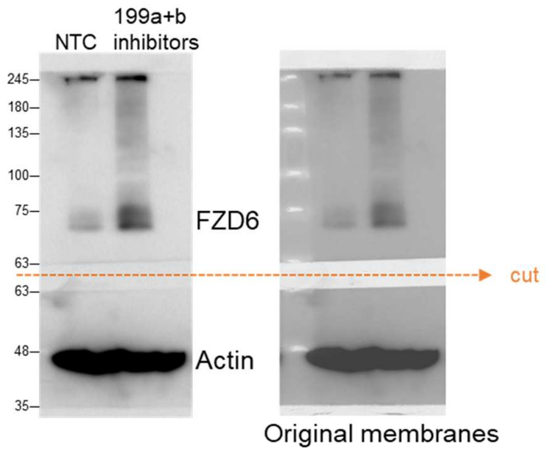

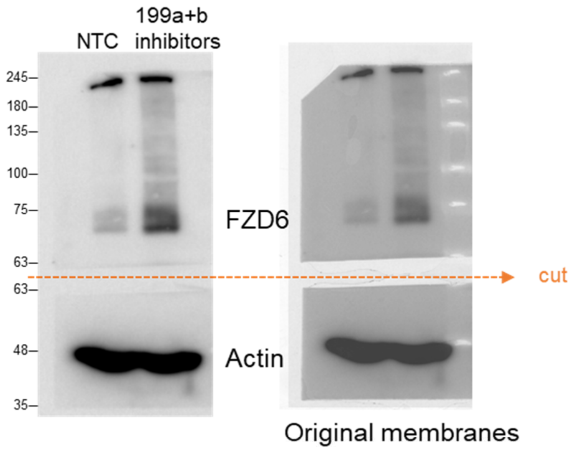

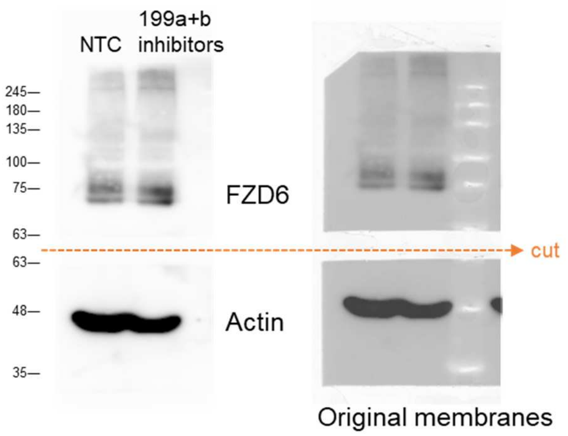

Original gels - Supplementary Figure 3B

Digital gel images were obtained using the GelDoc system.

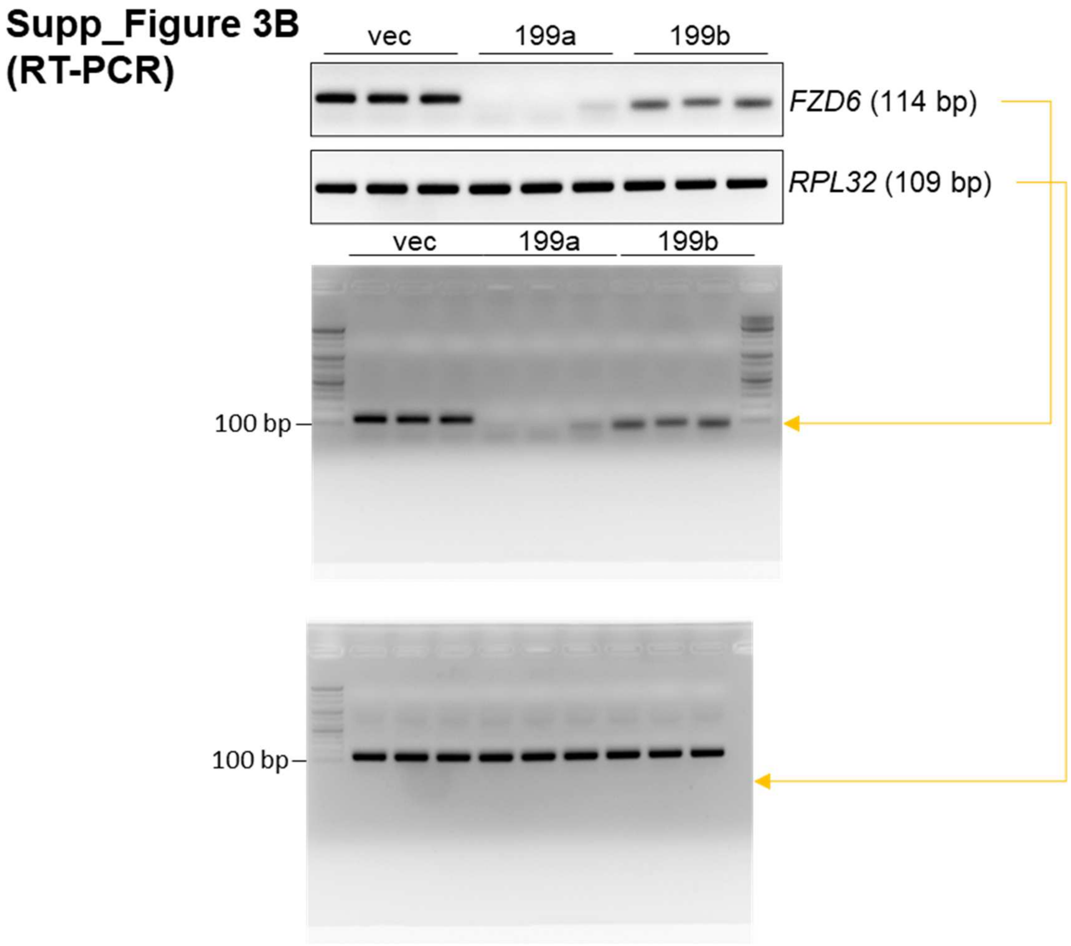

Original blots - Supplementary Figure 5B

The blots were cut prior to hybridization with antibodies.
Digital Western blot images were obtained using the ChemiDoc system.

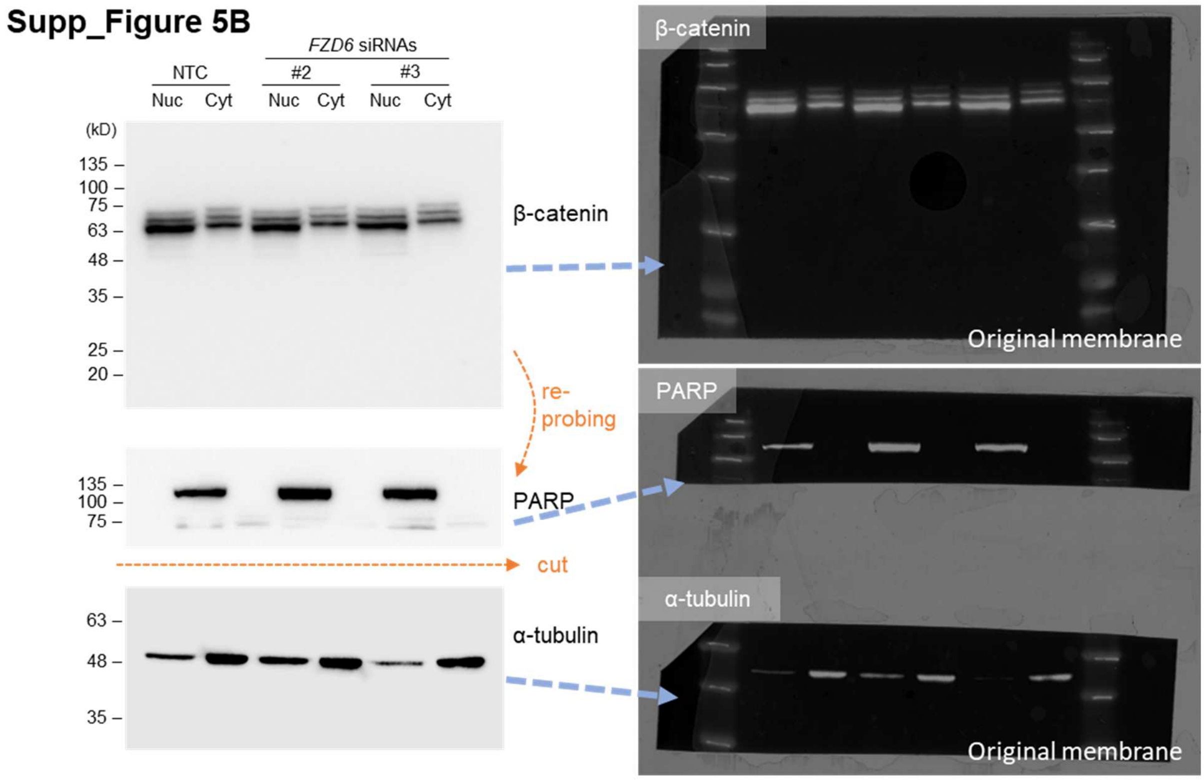

Supplement: Supplementary file 1 — Supplementary Information. [file 41598_2023_44716_MOESM1_ESM.pdf]
